# Weight Gain Prevention among Midlife Women: A Randomized Controlled Trial to Address Needs Related to the Physical and Social Environment

**DOI:** 10.3390/ijerph13060530

**Published:** 2016-05-25

**Authors:** Courtney D. Perry, Dennis Degeneffe, Cynthia Davey, Grace Kollannoor-Samuel, Marla Reicks

**Affiliations:** 1 Unity Point Health-Des Moines, 1200 Pleasant Street, Des Moines, IA 50309, USA; Courtney.Perry@unitypoint.org; 2 Consumer Centric Solutions, St. Paul, MN 55116, USA; ddegeneffe@comcast.net; 3 Clinical and Translational Science Institute, University of Minnesota, 717 Delaware St SE, 1-25, Minneapolis, MN 55414, USA; davey002@umn.edu; 4 Yale School of Public Health, 60 College Street, New Haven, CT 06510, USA; grace.kollannoorsamuel@yale.edu; 5 Department of Food Science and Nutrition, University of Minnesota, 1334 Eckles Ave, St. Paul, MN 55108, USA

**Keywords:** weight gain prevention, midlife, women, eating occasions, environment

## Abstract

Women tend to gain weight at midlife (40–60 years) increasing risk of obesity-related chronic diseases. Within specific eating occasions, needs related to the physical and social environment may result in less healthy eating behavior, which can lead to weight gain over time. The purpose of this study was to determine if a dietitian-delivered nutrition counseling intervention tailored to eating occasion needs could improve diet and prevent weight gain among midlife women over two years. A randomized controlled trial was conducted with healthy midlife women (*n* = 354) in one U.S. metropolitan area. The intervention group (*n* = 185) received ten hours of individual nutrition counseling from dietitians over six months, while women in a control group (*n* = 169) received no counseling. Measured height, weight and waist circumference, and dietary intakes were collected at baseline and every six months over two years. Mixed linear models were used to test for intervention effect on change in outcome variables over time. Dietary intakes of fruit, reduced/low-fat dairy foods and refined grains were significantly improved over time in the intervention compared to control group. However, the intervention had no effect on weight over time (*p* = 0.48). Nutrition counseling tailored to address eating occasion needs improved self-reported diet but did not significantly affect weight change.

## 1. Introduction

Several prospective and observational studies demonstrated that midlife women gained 0.61–0.87 kg/year based on 3 to 11 years of follow-up [1,2,3]. Weight gain during midlife has been associated with increased risk of stroke [4], prehypertension [5], some forms of cancer [6], and structural abnormalities of the knee [7]. Weight gain prevention may result in fewer negative health consequences and reduced expenditure for overweight/obesity treatment.

Interventions to prevent weight gain among adults have produced mixed results and have not consistently reported effects on dietary intake. A systematic review of interventions to prevent weight gain published in 2009 identified nine studies with adults [8], however, most involved adults at younger ages with five of nine producing significant weight differences between intervention and control groups at the end of one- to three-year follow-up periods. For example, among younger women (25–44 years), behavioral strategy sessions related to diet and physical activity were delivered in clinic-based groups or via a correspondence course, and resulted in no effect on weight change compared to a control group over three years [9]. In another study with younger women (mean age 40 years), group sessions on behavior change strategies resulted in weight loss in the intervention group and weight gain in the control group over a one-year period [10]. Among postmenopausal women, more than half (55%) of intervention participants in a behavioral diet and physical activity program were at or below baseline weight compared to 26% of control participants after 4.5 years; however, the program focused on achieving weight loss initially [11]. Several previous interventions designed to prevent weight gain did not report dietary changes [9,12,13]. The limited number of existing effective approaches among midlife women and inconsistent reporting of dietary changes suggests a need for careful examination of the efficacy of lifestyle interventions to prevent weight gain for women at midlife.

Social Cognitive Theory [14] proposes that people acquire behavioral patterns, such as dietary behaviors, based on the reciprocal influences of (1) the individual’s set of learned experiences; (2) behavioral responses to cues to achieve goals; and (3) the physical and social environment which provides context for behavioral patterns. Food choices made by midlife women during eating occasions at home and away from home are dependent on the physical environment (foods available and accessible), and the social environment (family, peers and others who may be present). For example, midlife women participating in a two-year weight loss intervention, identified the home environment as a critical influence on the maintenance of newly-developed healthier dietary habits [15].

Because factors in the physical and social environment that affect dietary behaviors vary for individual women, tailored intervention programs that meet personal weight control needs are recommended to achieve changes in eating and exercise behaviors [16,17]. A systematic review indicated that tailoring nutrition education for adults contributed to eating behavior change and maintenance over time [18]. Segmentation analysis has been used as the basis for previous tailored intervention studies. Researchers have divided consumers into groups based on shared characteristics [19,20], that affect food intake behaviors. Another less common but potentially useful approach is to categorize eating occasions (meals and snacks), as tested in a feasibility study among midlife women [21]. Results from this study showed that the type of need addressed within an eating occasion affected food and nutrient intakes among midlife women. Needs were based on the physical and social environment or situational context (who is present, time of day, day of the week, where consumption occurs, *etc.*). For example, “routine family meal” occasions were likely to be dinner meals eaten at home with family members. Needs for these occasions were based on meeting family expectations and were likely to have high energy and fat content. In contrast, “healthy regimen” occasions were more likely to be breakfast or lunch meals eaten alone at home on a weekday. Needs for these occasions were based on a desire to eat healthy and take care of oneself with low energy and fat content. Repeatedly experiencing eating occasions where food and nutrient intakes are less healthy in response to needs based on the physical and social environment may lead to weight gain over time. Therefore, an intervention program tailored to common needs within eating occasions may be effective in preventing weight gain.

The primary objective of this study was to determine if dietitian-delivered individual nutrition counseling tailored to common needs within eating occasions based on the physical and social environment could improve diet and prevent weight gain among midlife women. The study took place over a two-year period within a randomized controlled trial. The findings have relevance for modifying environments to better support healthy eating behavior among midlife women.

## 2. Experimental Methods

### 2.1. Participants and Recruitment

Women (40–60 years) were randomly selected from a market research firm database to represent the race/ethnicity distribution of the Twin Cities, Minnesota metropolitan area [22]. Sample size calculations indicated that for a 2-treatment, parallel-design study, 304 women would need to be enrolled. This number was based on an 80% probability that a 1-kg difference would be detected between groups, and on estimates of typical weight gain among midlife women [23,24]. Additional women were enrolled to compensate for a dropout rate of about 15%. During September–October 2008 (cohort 1) and March–April 2009 (cohort 2), the market research firm placed a screening call to potential participants. Inclusion screening criteria included: aged 40–60 years; no history of chronic disease; no physician-prescribed diet and not pregnant or breastfeeding.

**Figure 1:**
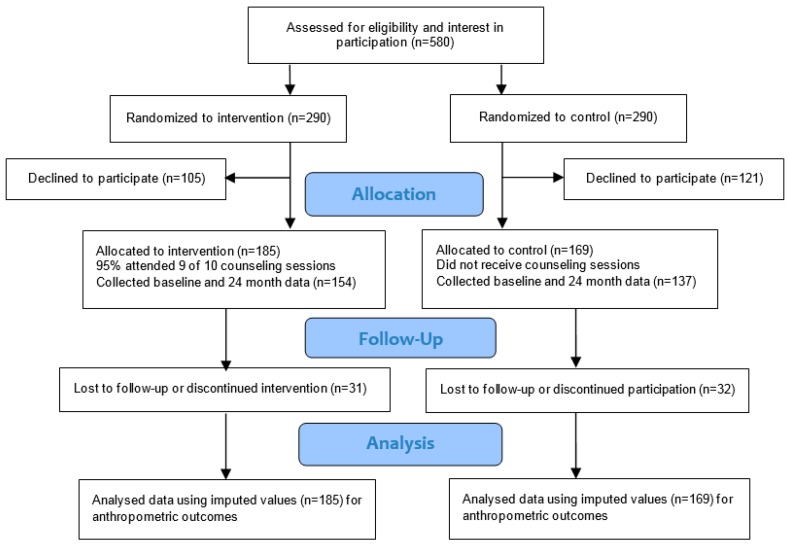
Flow diagram for midlife women participating in a weight gain prevention study by intervention and control group.

Women were assigned consecutively to the control or intervention group based on a stratified randomization procedure so that equal numbers of women in each group were employed full-time, had children <12 years, and had an annual household income ≤$40,000. Initial BMI was not considered in the screening or randomization process. If a woman met the inclusion criteria and expressed interest in participation (*n* = 580), the study coordinator called to schedule separate baseline/informational sessions for women in the intervention and control groups (Figure 1). The baseline/informational sessions were held for groups of about 10–15 women where study tasks were described, consent was obtained, and baseline outcome data were collected.

During the scheduling call, the intervention was described to women assigned to the intervention group as an approach to improve eating habits tailored to participant needs within eating occasions. Expectations regarding counseling sessions and follow up measurements over a two-year period were also described. Expectations for women assigned to the control group were described as collection of food records and measurement of weight only over a two-year period. Participants were not informed about the specific aims and randomized nature of the study to avoid having women in the control group initiate significant dietary and physical activity changes that could affect weight change over the two year period. Women were randomized into groups prior to providing information about study procedures because the extent of their expected involvement represented a significant long-term time commitment, therefore, awareness of the study procedures before attending a baseline information session was desirable. After this scheduling call, 121 women declined participation in the control group and 105 women declined participation in the intervention group.

All subjects gave their informed consent for inclusion before they participated in the study. The study was conducted in accordance with the Declaration of Helsinki, and the protocol was approved by the University of Minnesota Institutional Review Board (Project identification code: 0612S97611). Women in the intervention and control groups were compensated with US $200 and $100, respectively based on the amount of time expected for participation.

### 2.2. Intervention Overview

Women in the intervention group received ten hours of individualized counseling from a registered dietitian in 1-h sessions every two weeks over a 6-month period. Counseling was tailored to provide advice about healthful eating based on eating occasion needs based on the physical and social environment. Women in the control group did not participate in counseling sessions. All women attended a data collection session every six months over two years to have height, weight and waist circumference measurements recorded and to provide completed food record forms and other questionnaires. For these data collection sessions, women were asked to attend an individual session during a specific time in the morning before they ate breakfast (8:00–10:30 a.m.).

### 2.3. Intervention Procedures

#### Lesson Development and Implementation

A previous segmentation study based on 5556 eating occasions from a national sample of 1663 midlife women (40–60 years) identified and described six distinct categories of needs for eating occasions [25]. Descriptive names were assigned: healthy express, comforting interludes, indulgent escapes, nurturing family meals, sensible meals, and fast fueling (Table 1). Health-oriented eating occasions (healthy express, comforting interludes, and sensible meals) were characterized by lower fat and higher fruit and whole grain intakes, whereas occasions with less-healthy needs (indulgent escapes, fast fueling, and nurturing family meals) were highest in fat intake, and higher in energy, refined grain, and sucrose intakes [25]. The number of less-healthy eating occasions experienced was positively associated with BMI.

The research team including five registered dietitians developed learning objectives and instructional activities for three lessons for each eating occasion need category by applying Social Cognitive Theory constructs (Table 1) [14]. For example, food intake during “nurturing family meals” occasions may be influenced by social environmental needs to make dinner a family time with minimal complaints, and suppression of personal nutrition needs to meet food preferences of family members. In response, a lesson topic addressed the improvement of meal planning skills to include healthy foods the whole family liked, thus improving availability and accessibility of healthy foods for the participant. The lessons were interactive in nature, requiring participants to personalize content according to their physical and social environments and to complete goal-setting and progress-monitoring homework between lessons. Small, incremental, positive dietary changes were introduced and reinforced consistent with the small change approach recommended by others [26,27]. The lessons were focused solely on improving eating behaviors and not on weight loss, weight maintenance or promotion of physical activity. The goal was to determine if addressing common needs related to environmental factors within eating occasions that contribute to less healthful dietary intakes could prevent weight gain over time.

Registered dietitians conducted individualized counseling for women in the intervention group in homes, coffee shops or community locations in approximately 1-h sessions every two weeks for six months. The lessons were also personalized to each participant based on the most common needs they had within eating occasions, as determined by a classification tool designed for this study. The tool included a set of 20 statements to classify needs based on a previous study [25]. Women selected strongly agree/strongly disagree responses to the 20 statements regarding usual eating occasions (meals and snacks) over the previous week. The items were based on (1) needs within the eating occasion: “I wanted to…” (e.g., “treat myself” or “connect with others/family”) and (2) benefits sought in the food/beverages consumed: “I wanted something that…” (e.g., “is healthy to eat” or “is portable”). Values assigned to agree/disagree responses were entered into an algorithm to indicate the frequency with which women experienced the various needs within eating occasions.

### 2.4. Measures

At baseline, 6-, 12-, 18-, and 24-month sessions, research team members measured height, weight and waist circumference. The women completed a 3-day food record [28], the eating occasion classification tool, a leisure time exercise questionnaire and questions to assess menopausal status [29] and brought the completed materials to the sessions.

Data about frequency of strenuous, moderate, and mild physical activity were collected using a validated questionnaire developed by Godin and Shephard [30] to monitor changes in physical activity throughout the 2-year period. An activity score in arbitrary units was determined by summing the number of episodes of each type of activity multiplied by its assigned metabolic equivalent (9, 5, and 3 for strenuous, moderate, and mild activity, respectively).

Serving sizes for food groups were determined as indicated by NDSR and were grouped by combining similar foods according to United States Department of Agriculture MyPlate food groups [31]. Fruits included whole, canned, or dried fruit, 100% fruit juice, and avocado. Vegetables included all raw or cooked (not including fried) vegetables, potatoes and other starchy vegetables, legumes, and vegetable juice. Two grain groups (refined and whole grain) were represented by breads, crackers, pasta, cereals, cakes, cookies, snack bars, popcorn and chips. Two dairy groups (regular or reduced/no fat) included milk, cheese, and yogurt. Added fats included regular margarine, oil, shortening, butter and animal fats, salad dressings, and gravy.

#### Anthropometric Measurements

Height, weight, and waist circumference were measured using a standardized protocol by research team members trained according to standard procedures [32]. Height was measured barefoot using a stadiometer (Seca 202, Hanover, Maryland) to the nearest 0.1 centimeter (cm). Weight was measured barefoot and in light clothing (cloth shorts and a tee-shirt) on a digital scale to the nearest 0.1 kilogram (kg) (Tanita BWB-800P Digital Medical Scale, Arlington Heights, IL, USA). Scales were calibrated prior to use with standard weights (Mettler-Toledo Calibration weights, Columbus, OH, USA). Waist circumference was measured to the nearest 0.1 cm, with the waist measured under clothing at the narrowest point between the iliac crest and the lowest rib with a non-flexible tape measure. All measurements were done in the morning after an overnight fast, in triplicate with a mean calculated for analysis. BMI was calculated by dividing weight (kg) by height (m) squared.

### 2.5. Statistical Analysis

Descriptive statistics for demographic characteristics of participants include means and standard deviations (SD) for quantitative characteristics and number (%) for categorical characteristics. At baseline, between-group differences were assessed with chi-square tests for categorical demographic characteristics and with two-sample *t*-tests for age and household composition.

A multiple imputation method was used to replace missing data. A fully conditional model with linear regressions was used for imputing continuous variables. After imputing three to five data sets, at least 90% relative efficiency was obtained for each imputed variable. Estimates obtained from these imputed data sets were combined into a single overall estimate. Analyses of variance did not suggest significant group × time interaction in any outcome of interest. Therefore, group comparisons were not conducted at each time point.

Overall effect of intervention on the outcome was assessed using longitudinal mixed modeling. All participants with baseline data were included in the mixed models based on the multiple imputation method described. Mixed modeling for the primary outcomes (weight, BMI, and waist circumference) and secondary outcomes (dietary data from the food records) were conducted using longitudinal data from 354 and 322 participants, respectively. Initial mixed models included both fixed (group, time, group by time interaction, baseline outcome, quadratic and cubic time) and random (intercept, time, quadratic and cubic time) effects. The Toeplitz or Unstructured covariance structure, appropriate for equally spaced follow-ups was selected for each model based on the Akaike Information Criteria. Initial models did not include additional covariates as these covariates did not satisfy the criteria for confounding. Mixed effect model for each outcome was selected based on the Akaike Information Criteria. Thus, final models included both fixed (baseline outcome, group and time variable), and random effects (intercept, and time). In addition, all mixed models predicting weight or dietary intake variables were adjusted for education, income and race to account for bias associated with loss to follow-up. The mixed models examining the effect of intervention on dietary intake components were additionally adjusted for baseline body weight. Significance was set at *p* < 0.05 for all tests. Statistical Analysis Software (version 9.2, copyright 2002–2008; SAS Institute, Cary, NC, USA) was used for all statistical analyses.

## 3. Results

### 3.1. Retention

Women who did not have weight measured at baseline and 24 months were considered dropouts. Throughout the two-year period, 63 women (17.8%) dropped out of the study; for 48 women (13.6%) this occurred between baseline and 6 months (Figure 1). A similar percentage of women dropped out from each group (16.8% and 18.9% for the intervention and control groups, respectively). Reasons for dropping out included lack of time (*n* = 22), no reason reported (*n* = 14), personal reasons (*n* = 8), moved out of the area (*n* = 5), lack of interest (*n* = 4) and death (*n* = 1). Nine women could not be reached to determine a reason for dropping out. Women who dropped out of the study between baseline and six months tended to have a higher weight (*p* = 0.01), waist circumference (*p* = 0.007), and BMI (*p* = 0.03), lower levels of education (*p* = 0.02) and income (*p* = 0.0001), and were more likely to be nonwhite (*p* = 0.003) compared to women who completed the study.

### 3.2. Demographic Characteristics

Most study participants were non-Hispanic white, married and living with a spouse, employed full- or part-time, and had completed some college or had a college degree. Total household income for most women (84.5%) was >$40,000/year (Table 2). Groups were equivalent with respect to all baseline covariates except mean physical activity score (intervention *vs.* control group = 5.3 *vs.* 5.7; *p* = 0.046; data not shown). No significant differences were observed in menopausal status at baseline (Table 2) and no differences were observed between cohorts (data not shown).

### 3.3. Food Group and Nutrient Intakes

No changes were observed in energy or nutrient intakes over the 2-year period (Table 3). However, several changes were observed in food group intakes. Compared to the control group, the intervention group reported consuming significantly higher servings of fruits (mean difference = 0.18 (95% confidence interval = 0.02, 0.35); *p* = 0.03) and low/reduced fat dairy (mean difference = 0.14 (95% confidence interval = 0.04, 0.24); *p* = 0.01), and significantly lower servings of refined grains (mean difference = −0.32 (95% confidence interval = −0.57, −0.07); *p* = 0.01) (Table 3).

### 3.4. Weight Change

Mean baseline BMI was in the overweight range for both the intervention (28.0 kg/m^2^) and control groups (27.5 kg/m^2^; Table 4). Intervention had no effect on weight, waist circumference or BMI over time (*p* = 0.48, 0.55 and 0.90, respectively; Table 4). Women in the intervention group gained 0.2 kg over the two-year period (76.6 ± 1.3 (mean ± SE) to 76.8 ± 1.3), while women in the control group gained 0.4 kg (74.2 ± 1.4 to 74.6 ± 1.4). Waist circumference decreased by 0.3 cm in the intervention group (85.4 ± 1.0 to 85.1 ± 1.0) and by 0.1 cm in the control group (83.7 ± 1.0 to 83.6 ± 1.0). Over the two-year period, BMI increased by 0.1 unit in the intervention group (28.0 ± 0.5 to 28.1 ± 0.5) and by 0.6 units in the control group (27.5 ± 0.5 to 28.1 ± 0.5) (Table 4).

## 4. Discussion

An important goal of the 6-month individual nutrition counseling intervention was to prevent weight gain over a two year period of time. However, the longitudinal mixed modeling analysis did not identify a significant intervention effect on any of the primary anthropometric measurements after two years. Over the two years, both groups increased weight, with the intervention group having a smaller increase than the control group (0.1 *vs.* 0.2 kg/year, on average, respectively), with no differences between groups. Previous studies have documented larger weight gains in the range of 0.61–0.87 kg/year for midlife women based on 3 to 11 years of follow-up [1,2,3]; therefore, weight gain in the intervention and control groups in the current study was less than has been previously reported. While the control group did not receive individual counseling, weight and eating behaviors were monitored every 6 months over the 2-year period, which may have contributed to the lack of differences between groups. Accountability to oneself and others for weight change over time based on monitoring may provide motivation for weight loss or maintenance [33,34,35]. In addition, women in the control group may have been interested in weight gain prevention for many reasons and could have instituted lifestyle changes on their own during this period.

After counseling, women in the intervention group in the current study significantly decreased refined grain servings, and increased fruit intake and low/reduced-fat dairy servings compared to the control group. Women were encouraged to meet less-healthy eating occasion needs with healthier options available in the physical environment to improve dietary intake. For example, flavorful, fresh fruits were promoted instead of energy-dense foods to allow those experiencing “indulgent escapes” occasions to focus on the taste experience with healthier foods. If there was a need to eat something on the run during “fast fueling” occasions, women were encouraged to have healthy, convenient options available in the physical environment rather than easily accessible, less healthy foods. Previous studies have shown that improvements observed in the current study are consistent with dietary patterns that contribute to weight control [36,37]. Adherence to national dietary guidelines was also associated with successful aging among adults (49 years and older) defined as lacking disability and chronic disease [38]. However, dietary improvements in the current study did not impact changes in energy intake, which may be the most important predictor of weight control [39].

Other studies have shown that successful programs are based on tailored intervention approaches. Fruit and vegetable consumption was increased in veterans by using tailored messages and motivating interview calls [40]. Participants who perceived the messages as important, relevant to them, and applicable had a better response than those who did not receive tailored messages. Similarly, results from a systematic review showed that interventions tailored to participants had better outcomes than ones where participants were given generic information [41]. In the current study, a majority of women indicated in post-intervention satisfaction surveys that lesson topics were relevant and provided information they could use to make eating behavior changes. Therefore, while the intervention topics and counseling personalized to needs within eating occasions related to the physical and social environment may have been relevant and contributed to dietary improvements, the improvements were not of the magnitude necessary to differentiate weight change between groups.

The approach used in the current study was based on small changes over time to modify the physical and social environment in support of healthy food choices, which were introduced and reinforced over a six-month counseling period. However, the impact was not of sufficient magnitude to result in weight change differences between groups over two years. A recent systematic review of 11 weight gain prevention trials indicated that effective approaches may be based on changes in diet and physical activity and participation in group lifestyle sessions [42]. However, meaningful differences between groups that were also significant were only observed in about half of these studies indicating that the evidence supporting recommendations for specific strategies is limited.

### Limitations

Study limitations include using self-reported food records. Previous studies have shown that accuracy in self-reported food records and dietary recalls among women was related to social desirability, body size dissatisfaction, and fear of negative evaluation [43,44,45]. In the current study, efforts were made to improve accuracy by providing dietitian-delivered, in-person instructions on how to complete a food record booklet. An instructional DVD and pictures to use as guides were also provided. The majority of records were reviewed with the participant by a dietitian or a nutrition student trained in food record data collection to supplement information where it was lacking. However, the possibility remains that reporting biases could have influenced the results. The study aimed to enroll women by race/ethnic group proportions similar to census data from the Minneapolis/St. Paul metropolitan area. However, participants were more educated and had a higher income than the general population; therefore, results cannot be generalized to a broader group of midlife women. Potential selection bias due to high dropout rates after randomization is also a limitation.

## 5. Conclusions, Applications and Implications for Further Research

In summary, a dietitian-led individual nutrition counseling intervention, personalized to address eating occasion needs based on the physical and social environment improved dietary intake but did not prevent weight gain in a randomized controlled trial among midlife women. Dietitians may need to consider more intensive counseling focused on dietary behavior changes, monitoring of changes in weight and promotion of physical activity. Counseling tailored to needs has not been previously tested for effectiveness in preventing weight gain; therefore, this study provides additional information about the usefulness of this approach among midlife women.

## Figures and Tables

**Table 1:** Characteristics of needs within eating occasions and corresponding lessons delivered by dietitians to women in the intervention group.

Characteristics	Healthy Eating Occasion Needs			Less-Healthy Eating Occasion Needs		
	Healthy Express	Comforting Interludes	Sensible Meals	Nurturing Family Meals	Indulgent Escapes	Fast Fueling
Defining needs influenced by the physical and social environment	Eat healthy and quickly; Balance and control food & calorie intakes; Minimize time and effort	Personal moment, relax/unwind; Enjoy light meal or snack; Sensory gratification; Easy; Somewhat healthy, eat light	Eat healthy and responsibly; Control weight; Control calorie, fat, cholesterol, carbohydrate intakes	Show love, driven by emotional needs as caregiver; Family time with minimal complaints; Likely forsaking personal nutritional needs	Indulgent treat/reward; Focus on taste experience; Sensory gratification; Emotional benefit	Catch a quick bite, on the run; Eat quickly; Eat while doing other things; Dictated by time
Foods sought	Minimal effort, preparation, clean-up; Fast but still healthy; Low in fat, sodium; Nutrient dense	Satisfies craving; Fun to eat; Easy and fast, portable; Somewhat healthy	Healthy; Low in calories, fat, cholesterol, carbohydrates; Nutrient dense	Enjoyed by everyone; Nutritionally balanced; Within family budget	Satisfies cravings; Readily available; Treat, rich/indulgent; Easy to eat; Nostalgic connection	Minimal effort, planning, preparation, clean-up; Fast and ready to eat/portable; Easy to eat
Situation	Breakfast/lunch, weekday; Alone; At home or carried along	Breakfast/snack; Alone or with other adults; At home or away	Main meals; Alone or with other adults; At home	Dinner; Other adults and children; At home	Snacks; Alone; At home or restaurant	Lunch; Alone; At home or carried along
Lesson topics	Portion sizes; Time-saving strategies; Convenience foods; Nutrition information	Trigger identification, hunger scale use; Engage senses; Work/home environment changes; Alternative rewards	Health/nutrition claims on labels; Weekly meal plan; Recipe makeovers	Healthier options available; Meal planning; Grocery shopping tactics; Compare labels	Emotional *vs.* physical hunger, hunger scale; Non-food rewards; Compensation; Healthier options available; Strategies and plans	Healthy, on-the-go options, meal planning; Work/home environment changes; Nutritional cost of fast-food meals; Beverage calories

**Table 2:** Baseline demographic characteristics and weight status of all midlife women participating in a weight gain prevention study and by intervention and control group.

**Variable**	**All Participants *n* = 354**	**Intervention Group *n* = 185**	**Control Group *n* = 169**	***p* Value (*χ*^2^ Test)**
	***n* (%)**	***n* (%)**	***n* (%)**	
Race				0.64
White	275 (77.7 )	140 (75.7)	135 (79.9)	
Black	32 (9.0)	20 (10.8)	12 (7.1)	
Asian	18 (5.1)	9 (4.9)	9 (5.3)	
Native American	1 (0.3)	1 (0.5)	0 (0.0)	
Other	28 (7.9)	15 (8.1)	13 (7.7)	
Spanish Origin				0.99
Non-Hispanic	333 (94.1)	174 (94.1)	159 (94.1)	
Hispanic	21 (5.9)	11 (5.9)	10 (2.8)	
Marital Status				0.42
Never Married	29 (8.2)	17 (9.2)	12 (7.1)	
Married	272 (76.8)	136 (73.5)	136 (80.5)	
Married, not Living with Spouse	4 (1.1)	3 (1.6)	1 (0.6)	
Divorced/Widowed	49 (13.9)	29 (15.7)	20 (11.8)	
Income				0.88
<20 K	16 (4.5)	9 (4.9)	7 (4.1)	
20–40 K	39 (11.0)	24 (13.0)	15 (8.9)	
40–60 K	64 (18.1)	33 (17.8)	31 (18.3)	
60–80 K	61 (17.2)	31 (16.8)	30 (17.8)	
80–100 K	54 (15.3)	28 (15.1)	26 (15.4)	
>100 K	103 (29.1)	53 (28.6)	50 (29.6)	
Refused	17 (4.8)	7 (3.8)	10 (5.9)	
Employment				0.07
Full-time	171 (48.3)	85 (46.0)	86 (50.9)	
Part-time	104 (29.4)	48 (25.9)	56 (33.1)	
Not Employed	37 (10.4)	23 (12.4)	14 (8.3)	
Homemaker	36 (10.2)	4 (13.0)	12 (7.1)	
Retired	6 (1.7)	5 (2.7)	1 (0.6)	
Education				0.39
High School	25 (7.1)	13 (7.0)	12 (7.1)	
Some College	86 (24.3)	51 (27.6)	35 (20.7)	
2-Year College	62 (17.5)	35 (18.9)	27 (16.0)	
4-Year College	144 (40.7)	70 (37.8)	74 (43.8)	
Graduate School	37 (10.4)	16 (8.7)	21 (12.4)	
BMI Category				0.60
Less than 18.5	2 (0.7)	1 (0.6)	1 (0.7)	
18.5–24.9	116 (39.9)	60 (39.0)	56 (40.9)	
25.0–29.9	94 (32.3)	46 (29.9)	48 (35.0)	
30 and above	79 (27.1)	47 (30.5)	32 (23.4)	
Menopausal Status				0.67
Pre-menopausal	174 (49.2)	93 (50.3)	81 (47.9)	
Post-menopausal	180 (50.9)	92 (49.7)	88 (52.1)	
**Variable**	**Mean (SD)**	**Mean (SD)**	**Mean (SD)**	***p* Value (*t-*test)**
Age (years)	50.1 (5.1)	49.9 (5.1)	50.4 (5.1)	0.31
Household Design	Mean (SD)	Mean (SD)	Mean (SD)	
Adults in Household	2.2 (0.8)	2.2 (0.9)	2.2 (0.7)	0.99
Children in Household	0.9 (1.2)	1.0 (1.2)	0.8 (1.1)	0.16

**Table 3:** Energy, nutrient, and food group intake (means and standard errors ^a^) among all midlife women participating in a weight gain prevention study for the intervention and control groups over 2-years.

Dietary Variable ^b^	Baseline		6 Months		12 Months		18 Months		24 Months		Mean Difference	Overall Mixed Effect *p* Value
	Intervention (*n* = 174)	Control (*n* = 148)	Intervention (*n* = 167)	Control (*n* = 139)	Intervention (*n* = 152)	Control (*n* = 138)	Intervention (*n* = 151)	Control (*n* = 133)	Intervention (*n* = 154)	Control (*n* = 135)	Intervention and Control Group (95% CI) ^c^ (*n* = 322)	
Energy, kcal	1777 (38)	1744 (36)	1655 (33)	1726 (43)	1689 (39)	1666 (41)	1678 (42)	1658 (37)	1632 (37)	1696 (42)	−35.1 (−101.4, 31.1)	0.30
Total fat, g	39.8 (0.6)	39.2 (0.7)	38.9 (07)	39.1 (0.7)	38.3 (0.6)	37.5 0.7)	38.5 (0.7)	38.7 (0.8)	37.2 (0.6)	37.8 (0.8)	−0.25 (−1.33, 0.84)	0.67
Saturated fat, g	13.4 (0.3)	13.4 (0.3)	12.9 (0.3)	13.0 (0.3)	12.8 (0.3)	2.1 (0.3)	12.6 (0.3)	12.9 (0.4)	12.1 (0.3)	12.6 (0.3)	−0.03 (−0.50, 0.44)	0.90
Sodium, mg	1691 (27)	1668 (31)	1679 (25)	1608 (28)	1742 (29)	1778 (48)	1799 (34)	1674 (30)	1715 (31)	1694 (27)	32.5 (−19.0, 84.0)	0.21
Total fiber, g	10.6 (0.3)	11.1 (0.4)	12.1 (0.3)	11.9 (0.4)	11.4 (0.3)	11.6 (0.4)	11.6 (0.3)	11.5 (0.3)	11.8 (0.3)	11.6 (0.3)	0.51 (−0.26, 1.28)	0.19
Fruit ^d^	1.5 (0.1)	1.6 (0.1)	1.7 (0.1)	1.8 (0.1)	1.6 (0.1)	1.5 (0.1)	1.7 (0.1)	1.5 (0.1)	2.0 (0.1)	1.5 (0.1)	0.18 (0.02, 0.35)	0.03
Vegetable ^d^	2.6 (0.1)	2.8 (0.1)	2.9 (0.1)	3.1 (0.1)	2.9 (0.1)	2.8 (0.1)	2.8 (0.1)	2.9 (0.1)	2.8 (0.1)	3.0 (0.1)	0.03 (−0.19, 0.26)	0.78
Whole grains ^d^	1.2 (0.1)	1.3 (0.1)	1.4 (0.1)	1.3 (0.1)	1.5 (0.1)	1.4 (0.1)	1.3 (0.1)	1.2 (0.1)	1.2 (0.1)	1.3 (0.1)	0.10 (−0.05, 0.24)	0.21
Refined grains ^d^	3.8 (0.1)	3.6 (0.2)	3.1 (0.1)	3.3 (0.1)	2.9 (0.2)	3.4 (0.2)	3.1 (0.2)	3.2 (0.2)	3.0 (0.2)	3.3 (0.2)	−0.32 (−0.57, −0.07)	0.01
Low/reduced-fat dairy ^d^	1.1 (0.1)	1.1 (0.1)	1.2 (0.1)	1.1 (0.1)	1.3 (0.1)	1.0 (0.1)	1.2 (0.1)	1.1 (0.1)	1.1 (0.1)	1.1 (0.1)	0.14 (0.04, 0.24)	0.01
Sugars and candy ^d^	1.4 (0.1)	1.1 (0.1)	0.8 (0.1)	1.1 (0.1)	0.9 (0.1)	1.0 (0.1)	1.0 (0.1)	0.8 (0.1)	0.9 (0.1)	1.0 (0.1)	−0.14 (−0.32, 0.01)	0.08
Added fats ^d^	2.6 (0.1)	2.6 (0.1)	2.2 (0.1)	2.6 (0.1)	2.0 (0.1)	2.1 (0.1)	2.1 (0.1)	2.1 (0.1)	1.7 (0.1)	2.0 (0.1)	−0.18 (−0.38, 0.02)	0.07

^a^ Unadjusted means and standard errors; ^b^ Nutrients, but not food groups, are standardized to amount per 1000 kcal; ^c^ Results of longitudinal mixed modeling adjusted for baseline outcome (energy, total fat, saturated fat, sodium, total fiber, fruit, vegetable, whole grains, refined grains, low/reduced fat dairy, sugars and candy, added fats), baseline body weight, education, income, and race; ^d^ Serving sizes are based on the recommendations made by the Dietary Guidelines for Americans 2005 and the Food and Drug Administration.

**Table 4:** Anthropometric data (means and standard errors ^a^) for all midlife women participating in a weight gain prevention study and by subgroup in the intervention and control groups over 2-years.

Anthropometric Variable	Baseline		6 Months		12 Months		18 Months		24 Months		Mean Difference (95% CI) Intervention and Control ^b^	Overall Mixed Effect *p* Value
	Intervention	Control	Intervention	Control	Intervention	Control	Intervention	Control	Intervention	Control		
All women	(*n* = 185)	(*n* = 169)	(*n* = 169)	(*n* = 137)	(*n* = 156)	(*n* = 143)	(*n* = 152)	(*n* = 135)	(*n* = 154)	(*n* = 137)	(*n* = 354)	
Weight (kg)	76.6 (1.3)	74.2 (1.4)	76.1 (1.3)	74.2 (1.4)	76.5 (1.3)	74.4 (1.4)	76.3 (1.3)	74.2 (1.4)	76.8 (1.3)	74.6 (1.4)	−0.31 (−1.09, 0.46)	0.43
BMI (kg/m^2^)	28.0 (0.5)	27.5 (0.5)	27.8 (0.5)	27.5 (0.5)	27.9 (0.5)	27.6 (0.5)	27.9 (0.5)	27.5 (0.5)	28.1 (0.5)	28.1 (0.5)	−0.09 (−0.39, 0.21)	0.57
Waist circumference (cm)	85.4 (1.0)	83.7 (1.0)	84.5 (1.0)	83.2 (1.0)	85.2 (1.0)	83.6 (1.0)	85.2 (1.0)	83.3 (1.0)	85.1 (1.0)	83.6 (1.0)	−0.05 (−0.88, 0.77)	0.89

^a^ Unadjusted means and standard errors; ^b^ Results of longitudinal mixed modeling adjusted for baseline outcome (weight, BMI, or waist circumference), education, income and race.
